# Eliciting Renal Failure in Mosquitoes with a Small-Molecule Inhibitor of Inward-Rectifying Potassium Channels

**DOI:** 10.1371/journal.pone.0064905

**Published:** 2013-05-29

**Authors:** Rene Raphemot, Matthew F. Rouhier, Corey R. Hopkins, Rocco D. Gogliotti, Kimberly M. Lovell, Rebecca M. Hine, Dhairyasheel Ghosalkar, Anthony Longo, Klaus W. Beyenbach, Jerod S. Denton, Peter M. Piermarini

**Affiliations:** 1 Department of Anesthesiology, Vanderbilt University School of Medicine, Nashville, Tennessee, United States of America; 2 Department of Pharmacology, Vanderbilt University School of Medicine, Nashville, Tennessee, United States of America; 3 Department of Entomology, Ohio Agricultural Research and Development Center, The Ohio State University, Wooster, Ohio, United States of America; 4 Department of Chemistry, Vanderbilt University, Nashville, Tennessee, United States of America; 5 Vanderbilt Center for Neuroscience Drug Discovery, Vanderbilt University School of Medicine, Nashville, Tennessee, United States of America; 6 Department of Biomedical Sciences, Cornell University, Ithaca, New York, United States of America; 7 Institute of Chemical Biology, Vanderbilt University School of Medicine, Nashville, Tennessee, United States of America; 8 Institute for Global Health, Vanderbilt University School of Medicine, Nashville, Tennessee, United States of America; New Mexico State University, United States of America

## Abstract

Mosquito-borne diseases such as malaria and dengue fever take a large toll on global health. The primary chemical agents used for controlling mosquitoes are insecticides that target the nervous system. However, the emergence of resistance in mosquito populations is reducing the efficacy of available insecticides. The development of new insecticides is therefore urgent. Here we show that VU573, a small-molecule inhibitor of mammalian inward-rectifying potassium (Kir) channels, inhibits a Kir channel cloned from the renal (Malpighian) tubules of *Aedes aegypti* (*Ae*Kir1). Injection of VU573 into the hemolymph of adult female mosquitoes (*Ae. aegypti*) disrupts the production and excretion of urine in a manner consistent with channel block of *Ae*Kir1 and renders the mosquitoes incapacitated (flightless or dead) within 24 hours. Moreover, the toxicity of VU573 in mosquitoes (*Ae. aegypti*) is exacerbated when hemolymph potassium levels are elevated, suggesting that Kir channels are essential for maintenance of whole-animal potassium homeostasis. Our study demonstrates that renal failure is a promising mechanism of action for killing mosquitoes, and motivates the discovery of selective small-molecule inhibitors of mosquito Kir channels for use as insecticides.

## Introduction

Mosquitoes are vectors of debilitating diseases that take an immense toll on global health. Anopheline mosquitoes transmit pathogenic protozoans (*Plasmodium* sp.) that cause malaria. On an annual basis, there are an estimated hundreds of millions of episodes of malaria, which claim nearly one million lives; ∼85% of the victims are children under 5 years of age [1]. Culicine mosquitoes transmit viral pathogens that cause chikungunya, dengue, West Nile, and yellow fevers. Of the estimated 50–100 million individuals infected with dengue each year, hundreds of thousands require hospitalization and tens of thousands die [2]. These protozoan and viral pathogens are transmitted to humans solely by adult female mosquitoes, which feed on vertebrate blood to obtain nutrients for developing eggs.

The primary chemical agents currently in use for controlling mosquitoes are insecticides that target the nervous system. Although the development of insecticides such as DDT and pyrethroids, which modulate the activity of ion channels in the central nervous system of insects, offered promise for the eradication of mosquitoes in the 20th century, the emergence of resistance in mosquito populations has reduced their efficacy [3], [4]. Currently, there are not many alternatives, because no new insecticides for public-health use have been developed in over 30 years [5]. Thus, new chemicals and new approaches to control mosquitoes are urgently needed [5], [6].

A physiological process in the mosquito that has not yet been targeted by insecticides is the excretion of urine. The renal (Malpighian) tubules generate urine via the transepithelial secretion of NaCl, KCl, other solutes, and water from the extracellular fluid (hemolymph) to the tubule lumens [7], [8]. The tubules empty their secretions into the hindgut where solute and/or water is removed or added to the final urine before it is ejected via muscular contractions of the hindgut. Thus, inhibiting the function of Malpighian tubules—i.e., causing renal failure—is expected to disrupt extracellular fluid homeostasis with detrimental consequences to normal functions in the mosquito. Female mosquitoes would be particularly vulnerable to renal failure, because they would not be able to excrete the unwanted salt and water ingested during a blood meal [9], [10], [11].

The aim of the present study is to elicit renal failure in adult female mosquitoes (*Aedes aegypti*) using a small-molecule inhibitor of inward-rectifying potassium (Kir) channels. Kir channels are a phylogenetically ancient family of barium-sensitive K^+^ channels that play fundamental roles in nerve, muscle, endocrine, and epithelial function in diverse organisms [12]. In mosquito Malpighian tubules, Kir channels of the basolateral membrane are considered one of the two major routes for the uptake of K^+^ into the epithelium [13], [14]. The Malpighian tubules of *Ae. aegypti* express three cDNAs encoding Kir channel subunits (*Ae*Kir1, *Ae*Kir2B, *Ae*Kir3), which we have cloned and functionally characterized [13]. *Ae*Kir1 mediates robust K^+^ currents when expressed in *Xenopus* oocytes, whereas *Ae*Kir2B and *Ae*Kir3 produce relatively small and nominal K^+^ currents, respectively [13]. Thus, we focused on inhibiting *Ae*Kir1 in the present study.

## Results and Discussion

**Figure 1:**
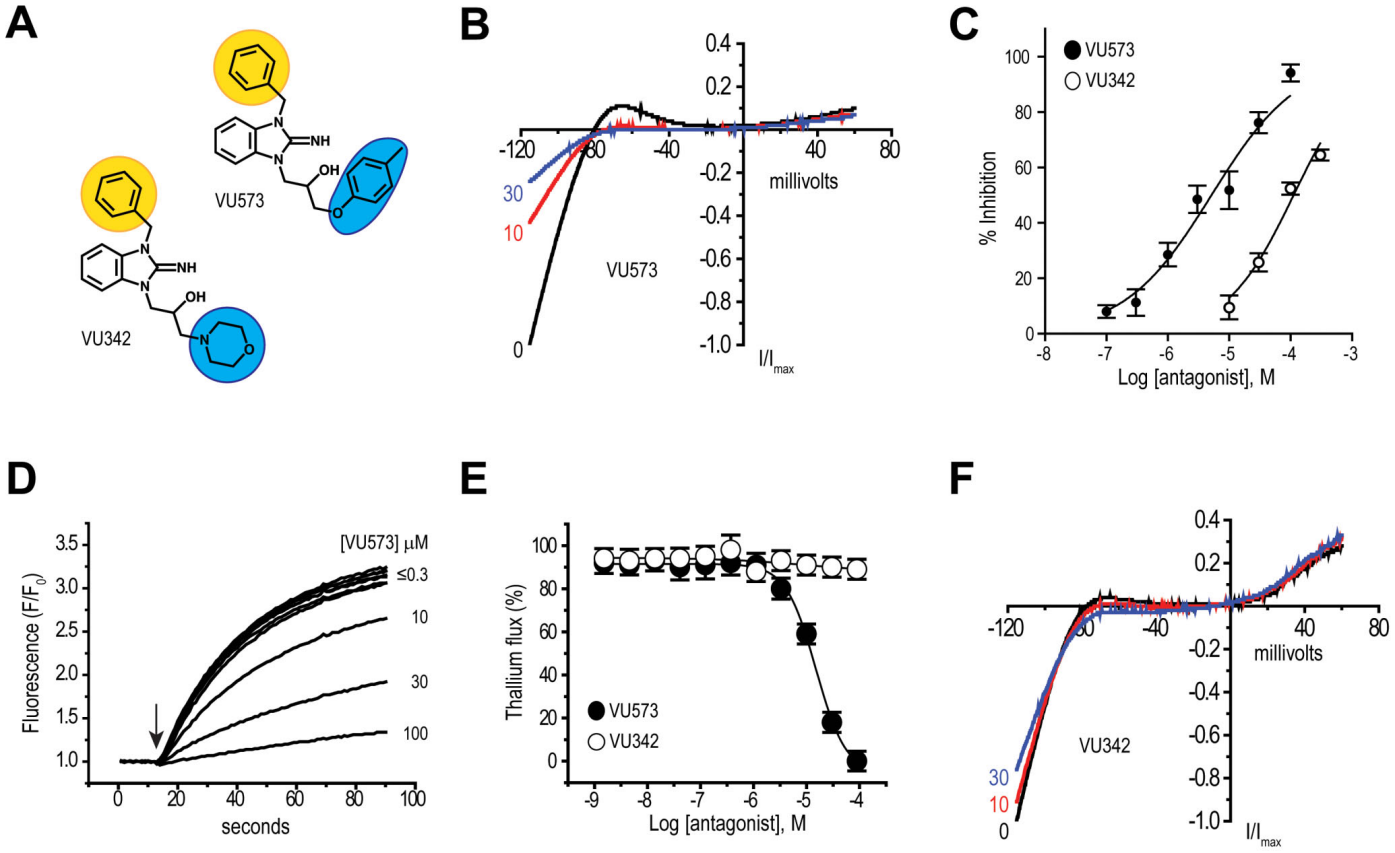
Small-molecule probes of *Ae*Kir1 expressed in T-REx-HEK-293 cells. (**A**) Chemical structures of the *Ae*Kir1 antagonist VU573 and inactive analog VU342. The ‘northern’ and ‘southern’ groups are indicated by yellow and blue shading, respectively. (**B**) Normalized *Ae*Kir1 current-voltage (I–V) relationships illustrating VU573-dependent inhibition at 0, 10, and 30 µM. Cells were voltage clamped at −75 mV and ramped between −120 mV and +60 mV. (**C**) Concentration-response curves of VU573 (filled circles) and VU342 (open circles) derived from patch clamp experiments (*n* = 4–9). The IC_50_ of VU573 and VU342 are 5.14±1.2 µM and 112±1.1 µM, respectively. (**D**) Dose-dependent inhibition of the *Ae*Kir1-mediated Tl^+^ flux by VU573 ranging in concentrations from ≤0.3 to 100 µM. The arrow indicates when Tl^+^ was added to the extracellular bath. (**E**) Concentration-response curves of VU573 (filled circles) and VU342 (open circles) derived from Tl^+^ flux assays. *n* = 2–3 independent experiments, each performed in triplicate. (**F**) Representative I–V relationships showing minimal effects of VU342 on the *Ae*Kir1-mediated currents at concentrations of 0, 10, and 30 µM. Values in panels **C** and **E** are means ± SEM.

With few exceptions, the small-molecule pharmacology of the Kir channel family is undeveloped [15]. In an effort to discover new modulators of human Kir1.1, we previously performed a high-throughput screen of approximately 225,000 small molecules from the National Institutes of Health Molecular Libraries Small-Molecule Repository [16]. The screen revealed compound VU573 (Figure 1A), which inhibits several human Kir channels [17]. Thus, we tested whether VU573 also inhibits the mosquito Kir channel, *Ae*Kir1.

The tetracycline-induced expression of *Ae*Kir1 in a human cell line (T-REx-HEK-293 cells) produces robust, barium-sensitive, inward-rectifying K^+^ currents in whole-cell patch clamp recordings (Figure S1). Bath application of VU573 inhibits the *Ae*Kir1-mediated currents in a dose-dependent manner (Figure 1B,C) with an IC_50_ of 5.14 µM (Figure 1C); this IC_50_ is similar to that observed for human Kir2.3, Kir3.x, and Kir7.1 [17].

To facilitate our search for derivatives of VU573 with an improved potency for *Ae*Kir1 over human Kir channels, we developed a high-throughput, fluorescence-based assay to measure *Ae*Kir1 activity in T-REx-HEK-293 cells. The assay uses thallium (Tl^+^) as a surrogate of K^+^ [15], [16], [17], [18]. As shown in Figure S2, the expression of *Ae*Kir1 in T-REx-HEK-293 cells mediates a robust flux of Tl^+^ when it is added to the extracellular medium. The flux is inhibited in a dose-dependent manner by VU573 with an IC_50_ of 15 µM (Figure 1D,E). The slightly higher IC_50_ of VU573 calculated via the Tl^+^-flux assay vs. patch clamp recordings is consistent with results of our previous study on the inhibition of human Kir2.3 and Kir7.1 channels by VU573 [17], and may reflect different channel permeabilities to Tl^+^ and K^+^ [19].

We used the Tl^+^-flux assay to assess the structure-activity relationships of 47 analogs of VU573 (Table S1), focusing on the ‘northern’ benzyl and ‘southern’ aryl-ether of VU573 (Figure 1A, Table S1) (see [17] for synthesis). Modification of the northern or the southern group of VU573 did not produce analogs with improved potency for *Ae*Kir1 (Table S1). However, the replacement of the southern aryl ether with a morpholine moiety produced compound VU342 (Figure 1A; Compound 9 in Table S1), which nominally inhibits the *Ae*Kir1-mediated Tl^+^ flux (IC_50_>100 µM; Figure 1E). When assessed in whole-cell patch-clamp experiments of T-REx-HEK-293 cells, VU342 is 22-times less potent than VU573 at inhibiting *Ae*Kir1 (IC_50_ = 112 µM; Figure 1C,F); the maximal inhibition of *Ae*Kir1 is only ∼65% and requires a concentration of 300 µM (Figure 1C). Accordingly, in subsequent experiments, we used VU342 as a negative control.

**Figure 2:**
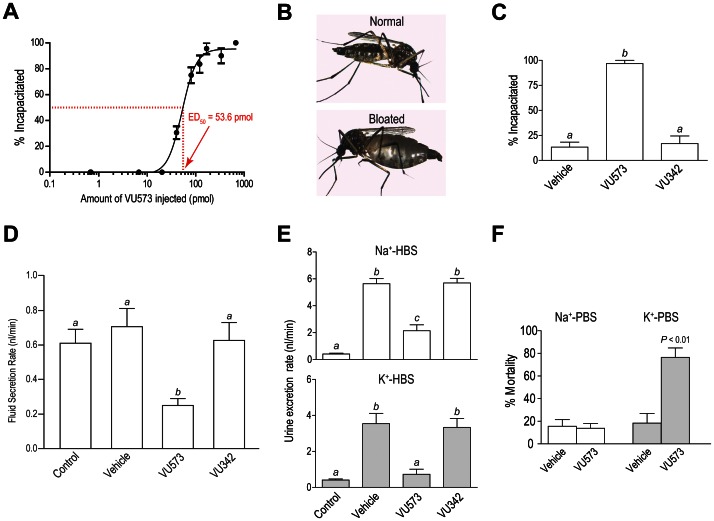
Effects of VU573 and VU342 on adult female mosquitoes (*Aedes aegypti*). (**A**) Dose-response curve of the incapacitating effects of VU573 on mosquitoes with an effective dose 50% (ED_50_) of 53.6 pmol. ‘% Incapacitated’ refers to the proportion of mosquitoes that are flightless or dead within 24 h after injection. Each mosquito was injected with 69 nl of PBS/15% DMSO containing an appropriate concentration of VU573 to deliver the dose indicated. The ED_50_ was determined by fitting a non-linear curve to the data (R^2^ = 0.95). *n* = 3–5 trials of 10 mosquitoes per dose. (**B**) Representative images of *Ae. aegypti* exhibiting ‘Normal’ and ‘Bloated’ abdomens. (**C**) Incapacitating effects resulting 24 h after injecting mosquitoes (69 nl each) with PBS containing the vehicle (15% DMSO), VU573 (10 mM), or VU342 (10 mM). *n* = 6 trials of 10 mosquitoes per treatment. Lower-case letters indicate statistical categorization of the means as determined by a one-way ANOVA and a Newman-Keuls posttest (*P*<0.05). (**D**) Effects of VU573 and VU342 on the in vitro secretion of fluid by isolated Malpighian tubules. Tubules were bathed in a peritubular Ringer solution (control) to which one of the following was added: vehicle (0.05% DMSO), VU573 (10 µM), or VU342 (10 µM). Secretion rates of the controls were calculated for the first 30 min, whereas those of the vehicle and small molecules were calculated 2 h after their addition. *n* = 7 tubules for control, vehicle, and VU342; *n* = 5 tubules for VU573. Lower-case letters indicate statistical categorization of the means as determined by a one-way ANOVA and a Newman-Keuls posttest (*P*<0.05). (**E**) Effects of VU573 and VU342 on the in vivo rates of urine excretion in intact mosquitoes. Mosquitoes were either uninjected (control) or injected with a Na^+^ or K^+^ HBS (900 nl) containing the vehicle (1.8% DMSO), VU573 (0.77 mM) or VU342 (0.77 mM). *n* = 8 trials of 3 mosquitoes for controls; *n* = 10 and *n* = 8 trials of 3 mosquitoes for Na^+^-HBS and K^+^-HBS, respectively. Lower-case letters indicate statistical categorization of the means as determined by a one-way ANOVA and a Newman-Keuls posttest (*P*<0.05). (**F**) Effects of injecting a Na^+^ or K^+^ PBS (900 nl) with the vehicle (1.1% DMSO) or VU573 (0.77 mM) on mosquito mortality. *n* = 5 trials of 10 mosquitoes each. Statistical differences between the vehicle and VU573 are determined with a paired t-test for each PBS. Values shown in all panels are means ± SEM.

We next assessed the effects of VU573 in adult female mosquitoes (*Ae. aegypti*). VU573 was dissolved in a phosphate-buffered saline (PBS) containing 15% DMSO and delivered directly to the hemolymph via intrathoracic microinjection (69 nl per mosquito). The injection of VU573 renders mosquitoes incapacitated (flightless or dead) within 24 hours in a dose-dependent manner (ED_50_ = 53.6 pmol; Figure 2A). Of the mosquitoes incapacitated by VU573, 92.3% were flightless while 7.7% were dead. Some of the incapacitated mosquitoes (3.6%) also exhibited greatly distended abdomens consistent with the retention of fluid in the absence of renal functions (‘bloated’ in Figure 2B). Parallel experiments in adult females of other important mosquito vectors (*Anopheles gambiae, Aedes albopictus, Culex pipiens*) show that injection of VU573 also incapacitates these mosquitoes (Figure S3).

To determine if the effects of VU573 on mosquitoes are associated with its inhibition of *Ae*Kir1, we compared the efficacy of VU573 to incapacitate mosquitoes (*Ae. aegypti*) with that of the inactive analog VU342. Figure 2C shows that nearly 100% of the mosquitoes injected with VU573, but less than 20% of those injected with the vehicle or VU342, are incapacitated within 24 hours. Furthermore, 10% of the mosquitoes injected with VU573 were bloated (e.g., Figure 2B), while none of the vehicle or VU342-injected mosquitoes retained fluid. Thus, the incapacitation of mosquitoes by VU573 likely arises from the inhibition of Kir channels—presumably those in the renal excretory system.

To assess whether VU573 inhibits the production of urine by Malpighian tubules we used the method of Ramsay [20]. As shown in Figure 2D, isolated Malpighian tubules bathed in a high-potassium Ringer solution (control) spontaneously secrete fluid at a rate of ∼0.6 nl/min in the first 30 min. The addition of VU573 to the peritubular bath (10 µM final concentration) significantly inhibits the rate of fluid secretion to 0.26 nl/min after 2 hours (Figure 2D), whereas the addition of the vehicle (0.05% DMSO) or VU342 (10 µM) has no effect on the rate of fluid secretion after 2 hours (Figure 2D). Thus, VU573 inhibits the first step in urine formation at the level of the Malpighian tubules.

To confirm the inhibition of Kir channels by VU573, we used two-electrode voltage clamping to measure the basolateral membrane voltage (V_bl_) and input resistance (R_pc_) of principal cells of isolated Malpighian tubules [21]. Peritubular application of VU573 (10 µM final concentration) significantly hyperpolarizes the V_bl_ by 7.0 mV while increasing the R_pc_ by 5.7 kΩ (Table 1); these changes are consistent with the blockade of Kir channels in the basolateral membrane of Malpighian tubules [21]. By comparison, peritubular application of barium at 5 mM, which is a generic blocker of potassium channels including Kir channels (e.g., Figure S1), also significantly hyperpolarizes the V_bl_ while increasing the R_pc_. The channel block by Ba^2+^ is significantly greater than that of VU573, which is to be expected given that it is less selective than VU573 (Table 1).

**Table 1:** Effects of VU573 (10 µM) and barium (5 mM) on the basolateral membrane voltage (V_bl_) and input resistance (R_pc_) of principal cells in isolated Malpighian tubules.

	V_bl_ (mV)	R_pc_ (kΩ)
Control (*n* = 5*)	−45.4±3.6	249.6±15.2
VU573 (*n* = 5)	−52.4±4.5 b	255.3±15.4 a
Control (*n* = 6)	−47.4±4.2	199.2±30.4
Barium (*n* = 6)	−59.3±3.3 c	322.8±31.0 d

a,b,c,d indicate *P*<0.02, 0.003, 0.008, and 0.0003, respectively (paired t-test).

Values are means ± SEM.

* *n* = number of principal cells impaled from as many isolated tubules.

To determine whether the VU573-mediated inhibition of fluid secretion by isolated Malpighian tubules causes renal failure in intact mosquitoes, we measured urine excretion rates using a method modified from the laboratory of Hansen [22]. Mosquitoes fed on a sucrose solution ad libitum (control) excrete urine at a rate of 0.41 nl/min (Figure 2E, top), whereas those injected with 900 nl of a Na^+^-HEPES-buffered saline (HBS)—a volume 30% less than that ingested with a blood meal [9]—excrete urine at a significantly higher rate of 5.64 nl/min (‘vehicle’ in Figure 2E, top). The rate of urine excretion is significantly dampened to 2.14 nl/min if the Na^+^-HBS contains VU573 (0.77 mM), whereas the rate is unaffected (5.7 nl/min) if the Na^+^-HBS contains VU342 (Figure 2E, top).

A more pronounced inhibition of urine excretion by VU573 is observed when 900 nl of a K^+^-HBS is injected (Figure 2E, bottom). Mosquitoes injected with the K^+^-HBS alone (vehicle) excrete urine at a significantly higher rate (3.54 nl/min) than the controls (Figure 2E, bottom), but if the injected K^+^-HBS contains VU573 (0.77 mM) then the rate of urine excretion is markedly reduced to 0.73 nl/min (Figure 2E, bottom). Urine excretion is unaffected if the K^+^-HBS contains VU342 (Figure 2E, bottom). Thus, VU573 inhibits both the production of urine in Malpighian tubules in vitro (Figure 2D) and the excretion of urine in vivo (Figure 2E). The more effective inhibition of urine excretion by VU573 in the presence of elevated hemolymph K^+^ is expected with the block of Kir channels in Malpighian tubules [13]. Taken together, the above findings suggest that VU573 incapacitates mosquitoes by interfering with K^+^ homeostasis.

In view of the above results, we sought to determine if the incapacitating effects of VU573 are enhanced by K^+^ in *Ae. aegypti*. In the control experiment, the injection of 900 nl of Na^+^-PBS with VU573 (0.77 mM) into the thoracic hemolymph of mosquitoes does not significantly increase mortality compared to those injected with the Na^+^-PBS vehicle alone (Figure 2F), but still renders them flightless within 24 hours. In contrast, the injection of K^+^-PBS with VU573 (0.77 mM) significantly increases mortality compared to the vehicle (Figure 2F). This finding mirrors the more pronounced effects of VU573 on the urine excretion rates of mosquitoes injected with K^+^-HBS vs. Na^+^-HBS (Figure 2E), suggesting that the lethal effects of VU573 stem from the disruption of hemolymph K^+^ homeostasis. Accordingly, the above findings indicate that VU573 would be most toxic to female mosquitoes after feeding on vertebrate blood, which presents a K^+^ load to the hemolymph [9], [10], [11]. In *Ae. aegypti*, about 30 min after feeding, the digestion of blood cells releases as much K^+^ for absorption into the hemolymph as that in the injection of K^+^-PBS [9]. Thus, soon after feeding on blood, a female mosquito is expected to succumb to the effects of VU573, which are amplified by elevated hemolymph K^+^ levels.

In conclusion, we have demonstrated that a small-molecule inhibitor of Kir channels elicits renal failure in female mosquitoes, which would decrease their reproductive output and ability to transmit pathogens by limiting the number of vertebrate blood meals they could consume. Therefore, such inhibitors could be considered as a potential new class of insecticides to be further developed for combatting the emerging problem of insecticide resistance in mosquitoes. The challenges that lay ahead are the development of: 1) small molecules that inhibit Kir channels of mosquitoes with greater potency than those of humans and beneficial insects, and 2) an efficient and effective system to deliver the inhibitors to mosquitoes. The high-throughput screening assay for *Ae*Kir1 established in the present study will expedite the former effort.

## Materials and Methods

### Expression vectors and sub-cloning

The open-reading frame of the *Ae*Kir1 cDNA cloned from Malpighian tubules of adult female *Ae. aegypti* (Genbank Accession #JQ753065 ) [13] was subcloned into a pcDNA5/TO expression vector (Invitrogen, Carlsbad, CA) using BamHI and XbaI restriction sites, using the following PCR primer pair : primer 1 = 5′-ACATTTCGAGTGACATTTGGGATCC GCCACCATGACAAAACTCTTCGAAGACTCC-3′; primer 2 = 5′-GTTTACAGTTTACAGTTTACATCTAGACTACAGTTCATCAACGAGTTCC-3′. The accuracy of the resulting *Ae*Kir1-pcDNA5/TO vector was confirmed by sequencing it in both the 5′ and 3′ directions.

### Stable cell line generation

Stably transfected polyclonal T-REx-HEK293 cell lines expressing *Ae*Kir1 under the control of a tetracycline-inducible promoter were established as described previously [16], [23]. Monoclonal cell lines were isolated through limiting dilution in 384-well plates and tested for tetracycline inducible Tl^+^ flux, as described below. T-REx-HEK293 lines were cultured in DMEM growth medium containing 10% FBS, 50 U/ml Penicillin, 50 µg/ml Streptomycin, 5 µg/ml Blasticidin S and 250 µg/ml Hygromycin.

### Whole-cell patch clamp electrophysiology

T-REx-HEK293-*Ae*Kir1 cells were voltage clamped in the whole-cell configuration of the patch clamp technique after overnight induction with tetracycline (1 µg/ml). Patch electrodes were pulled from silanized 1.5 mm outer diameter borosilicate microhematocrit tubes using a Narishige PP-830 two-stage puller. Electrode resistance ranged from 2.5 to 3.5 MΩ when filled with the following intracellular solution (in mM): 135 KCl, 2 MgCl_2_, 1 EGTA, 10 HEPES free acid, 2 Na_2_ATP (Roche, Indianapolis, IN), pH 7.3, 275 mOsm. The standard bath solution contained (in mM): 135 NaCl, 5 KCl, 2 CaCl_2_, 1 MgCl_2_, 5 glucose, 10 HEPES free acid, pH 7.4, 290 mOsm. The high-K^+^ bath contained (in mM): 90 NaCl, 50 KCl, 2 CaCl_2_, 1 MgCl_2_, 5 glucose, and 10 HEPES-free acid, pH 7.4, 290 mOsmol. Whole-cell currents were recorded under voltage-clamp conditions using an Axopatch 200B amplifier (Molecular Devices, Sunnyvale, CA). Electrical connections to the amplifier were made using Ag/AgCl wires and 3 M KCl/agar bridges. Electrophysiological data were collected at 5 kHz and filtered at 1 kHz. Data acquisition and analysis were performed using pClamp 9.2 software (Axon Instruments). After achieving stable whole-cell currents, VU573 or VU342 was applied intermittently or continuously for 2 to 10 min, followed by application of 2 mM BaCl_2_. All recordings were made at room temperature (20–23°C).

### Test compound and stimulus plate preparation

Compound master antagonist plates were created by serial diluting compounds 1∶3 from 30 mM stocks in 100% DMSO using the BRAVO liquid handler (Agilent Technologies, Santa Clara, CA). Assay daughter plates were created using the ECHO 555 liquid hander (Labcyte, Sunnyvale, CA), transferring 240 nl from the master plate to the daughter plate for each well followed by addition of 40 µl of assay buffer resulting in antagonist compound concentration response curves starting at 200 µM (2× final concentration). Tl^+^ stimulus buffer contained (in mM): 125 mM sodium bicarbonate (added fresh the morning of the experiment), 1 mM magnesium sulfate, 1.8 mM calcium sulfate, 5 mM glucose, 12 mM Tl^+^ sulfate, and 10 mM HEPES, pH 7.3 at 5× the final concentration to be assayed.

### Thallium flux assays

Cells were loaded with the Tl^+^ sensitive fluorescent dye FluoZin-2( acetoxymethyl ester form) and plated in clear-bottom 384-well plates essentially as described previously [16], [18]. Cell plates and daughter compound plates were loaded onto a kinetic imaging plate reader (FDSS 6000; Hamamatsu Corporation, Bridgewater, NJ). All recordings were made at room temperature (20–23°C). Appropriate baseline readings were taken (10 images at 1 Hz; excitation, 470±20 nm; emission, 540±30 nm) and 20 µl test compounds were added followed by 50 images at 1 Hz additional baseline. Following a 20 minute incubation period, baseline readings were taken for 10 seconds followed by addition of 10 µl of Tl^+^ stimulus buffer. An additional 240 images were taken at 1 Hz.

### Chemical synthesis

The methods for synthesizing VU573, VU342, and other analogs are described in detail elsewhere [17].

### Mosquito colonies

The following reagents were obtained through the MR4 as part of the BEI Resources Repository, NIAID, NIH: *Aedes aegypti* LVP-IB12, MRA-735, deposited by M.Q. Benedict, and *Aedes albopictus* ALBOPICTUS, MRA-804, deposited by Sandra Allan. Eggs from both *Aedes* species were raised to adults as described previously [24]. Adult female mosquitoes of *Anopheles gambiae* (Mbita strain) and *Culex pipiens* (Buckeye strain) were provided by the laboratories of Drs. Woody A. Foster and David L. Denlinger, respectively (the Ohio State University). For all experiments described below, only adult females of 3–10 days post-emergence were used.

### Mosquito toxicology experiments

Mosquitoes were first anesthetized on ice and then injected with 69 nl of fluid (see below) using a pulled-glass capillary attached to a nanoliter injector (Nanoject II, Drummond Scientific Company, Broomall, PA). The injected fluid was a sodium-based phosphate-buffered saline (Na^+^-PBS) containing 15% DMSO and various concentrations of VU573 or VU342 to deliver the doses indicated in Figures 2A, 2C, and S3. The Na^+^-PBS consisted of the following in mM: 137 NaCl, 2.7 KCl, 10 Na_2_HPO_4_, and 2 KH_2_PO_4_ (pH 7.5). After injection, the mosquitoes were placed in a small cage (10 females per cage) within a rearing chamber (28°C, 80% relative humidity, 12∶12 light∶dark) and allowed free access to a solution of 10% sucrose. The mosquitoes were observed 24 h after injection.

A similar approach was used to determine the toxicity of VU573 after a stress to hemolymph Na^+^ or K^+^ homeostasis (Figure 2F). However, in these experiments each mosquito was injected with 900 nl of fluid (100 nl/s) and the mosquitoes were not given access to sucrose. The injected fluid was a Na^+^-PBS or K^+^-PBS containing 1.1% DMSO and 0.77 mM of VU573. Vehicle controls received the respective PBS with DMSO alone. The K^+^-PBS consisted of the following in mM: 2.7 NaCl, 137 KCl, 2 Na_2_HPO_4_, and 10 KH_2_PO_4_ (pH 7.5).

### Isolated Malpighian tubule experiments

#### Fluid secretion assays

Fluid secretion rates from isolated Malpighian tubules (*Ae. aegypti*) were measured in vitro using the method described in [25], which is modified from that of Ramsay [20]. In brief, isolated tubules were bathed in a 50 µl drop of a mosquito Ringer solution with elevated K^+^ (see composition below), which was then covered with light mineral oil. A glass hook was used to pull the open proximal end of the tubule into the mineral oil where fluid was secreted. The diameter of the droplet was measured to calculate the secreted volumes.

In one set of tubules, the initial spontaneous rate of fluid secretion was calculated during the first 30 min (control). In another set of tubules, the rates of fluid secretion were determined 2 h after adding one of the following to the peritubular bath: the vehicle (0.05% DMSO), VU573 (10 µM), or VU342 (10 µM). The Ringer solution consisted of the following in mM: 119.4 NaCl, 34 KCl, 25 HEPES, 1.8 NaHCO_3_, 1.7 CaCl_2_, and 1.0 MgSO_4_, pH 7.1.

#### Electrophysiology

The basolateral membrane voltage (V_bl_) and input resistance (R_pc_) of principal cells were measured in isolated Malpighian tubules using two-electrode voltage clamping, as described previously [21]. In each experiment, a single principal cell near the distal (blind) end of the tubule was impaled with two glass microelectrodes: one measured V_bl_ and the other injected current (I_m_). The R_pc_ of the impaled cell was calculated from current-voltage plots that were generated via voltage clamping (see ref. [25] for details). After recording a steady-state V_bl_ and R_pc_ (control) from the impaled cell, either VU573 (10 µM) or BaCl_2_ (5 mM) was added to the peritubular bath. The resulting V_bl_ and R_pc_ (treatment) were measured again upon reaching a new steady-state (usually 1–2 min later).

### Mosquito excretion experiments

Urine excretion rates from intact mosquitoes (*Ae. aegypti*) were measured using a method modified from the laboratory of Hansen [22]. After anesthetizing mosquitoes on ice they were injected as described above with 900 nl of fluid (100 nl/s). The injected fluid was one of two HEPES-buffered saline (HBS) solutions containing 1.8% DMSO with 0.77 mM VU573 or 0.77 mM VU342. Vehicle controls received the respective HBS with DMSO alone. The Na^+^-HBS consisted of the following in mM: 146 NaCl, 4.2 *N*-methyl-D-glucammonium (NMDG)-Cl, and 25 mM HEPES (pH 7.5). The K^+^-HBS consisted of the following in mM: 10 mM NaCl, 75 mM KCl, 65.2 mM NMDG-Cl, and 25 mM HEPES (pH 7.5). After injection, mosquitoes were placed immediately in a graduated, packed-cell volume tube (MidSci, St. Louis, MO; 3 mosquitoes per tube) at room temperature. The mosquitoes were removed from the tubes with forceps after 2 h and the excreted urine was centrifuged into the graduated column of the tube for measurement. Uninjected control mosquitoes were handled exactly as above without the injection step.

### Statistical analyses

#### Tl^+^-flux assays

Data were analyzed using Excel (Microsoft Corp, Redmond, WA). Raw data were opened in Excel and each data point in a given trace was divided by the first data point from that trace (static ratio). The slope of the fluorescence increase beginning 5 s after Tl^+^ addition and ending 15 s after Tl^+^ addition was calculated. The data were then plotted in Prism software (GraphPad Software, San Diego, CA) to generate concentration-response curves after correcting for the slope values determined for baseline waveforms generated in the presence of vehicle controls. Potencies were calculated from fits using a four parameter logistic equation.

#### Mosquito toxicology and urine excretion

Data were analyzed using Prism 5 for Windows (Graphpad Software). To generate a dose-response curve for VU573, the doses (x-axis) were log transformed and then a non-linear curve was fitted to the data using the ‘log(agonist) vs. response’ algorithm. An ED_50_ was calculated from this curve. To compare 1) the incapacitating effects among the vehicle, VU573, and VU342 treatments, and 2) the rates of urine excretion among the control, vehicle, VU573, and VU342 treatments, one-way ANOVAs were performed with Newman-Keuls posttests. To compare the incapacitating effects between the vehicle and VU573 within each mosquito species, a paired t-test was used.

#### Malpighian tubule experiments

Data were analyzed using Prism 5 for Windows (Graphpad Software) and Excel (Microsoft Corp). To compare the rates of fluid secretion among control, vehicle, VU573, and VU342 treatments, a one-way ANOVA was performed with a Newman-Keuls posttest. To compare the V_bl_ and R_pc_ values of principal cells between control and treatment periods a paired t-test was performed.

## Supporting Information

Figure S1 Functional expression of *Ae*Kir1 in T-REx-HEK293 cells and its inhibition by barium (Ba^2+^). (**A**) Representative current traces recorded from stably transfected cells cultured overnight in the presence of tetracycline to induce channel expression of *Ae*Kir1. Recordings were made from a cell superfused with 5 mM K^+^ (top panel), 50 mM K^+^ (middle panel), or the control blocker 2 mM Ba^2+^ in 50 mM K^+^ (bottom panel). (**B**) Current (I) -voltage (V) relationships for *Ae*Kir1 bathed in 5 mM K^+^ (dark circle), 50 mM K^+^ (grey circle), or 50 mM K^+^ plus 2 mM Ba^2+^ (white circle). *n* = 3–7. (**C**) Concentration-response curve of Ba^2+^-dependent inhibition of *Ae*Kir1 with a 50% inhibition concentration (IC_50_) of 10 µM. Data are means ± SEM (*n* = 4–6).
(TIF)

Figure S2 Representative thallium(Tl^+^)-flux assay in T-REx-HEK293 cells loaded with the FluoZin-2 dye, which fluoresces (F/F_0_) in the presence of intracellular Tl^+^. Cells were cultured overnight with tetracycline (+Tet) to induce expression of *Ae*Kir1. Cells cultured without tetracycline (−Tet) served as controls . The arrow indicates when Tl^+^ was added to the extracellular bath.
(TIF)

Figure S3 Incapacitating effects of VU573 in three species of mosquitoes. Adult female mosquitoes were injected with Na^+^- PBS (69 nl) containing the vehicle (15% DMSO) or VU573 (10 mM). ‘% Incapacitated’ refers to the proportion of mosquitoes that are flightless or dead within 24 h after injection. Values are means ± SEM (*n* = 4 independent trials of 10 mosquitoes). Statistical differences between vehicle and VU573-treated mosquitoes were determined by a paired t-test for each species. *Anopheles gambiae* is the primary vector of malaria; *Aedes albopictus* is a vector of emerging arboviruses, such as dengue and Chikungunya fevers; *Culex pipiens* is a vector of West Nile virus and lymphatic filariasis.
(TIF)

Table S1 Structure-activity relationships for VU573 and its analogs. Values are means ± SEM (*n* = 1–3 independent Tl^+^ flux experiments in triplicate).
(DOC)
