# Supplementary figures and images for: Eliciting Renal Failure in Mosquitoes with a Small-Molecule Inhibitor of Inward-Rectifying Potassium Channels
Source: PLoS One. 2013 May 29;8(5):e64905. doi: 10.1371/journal.pone.0064905 (PMC3666979; doi:10.1371/journal.pone.0064905)

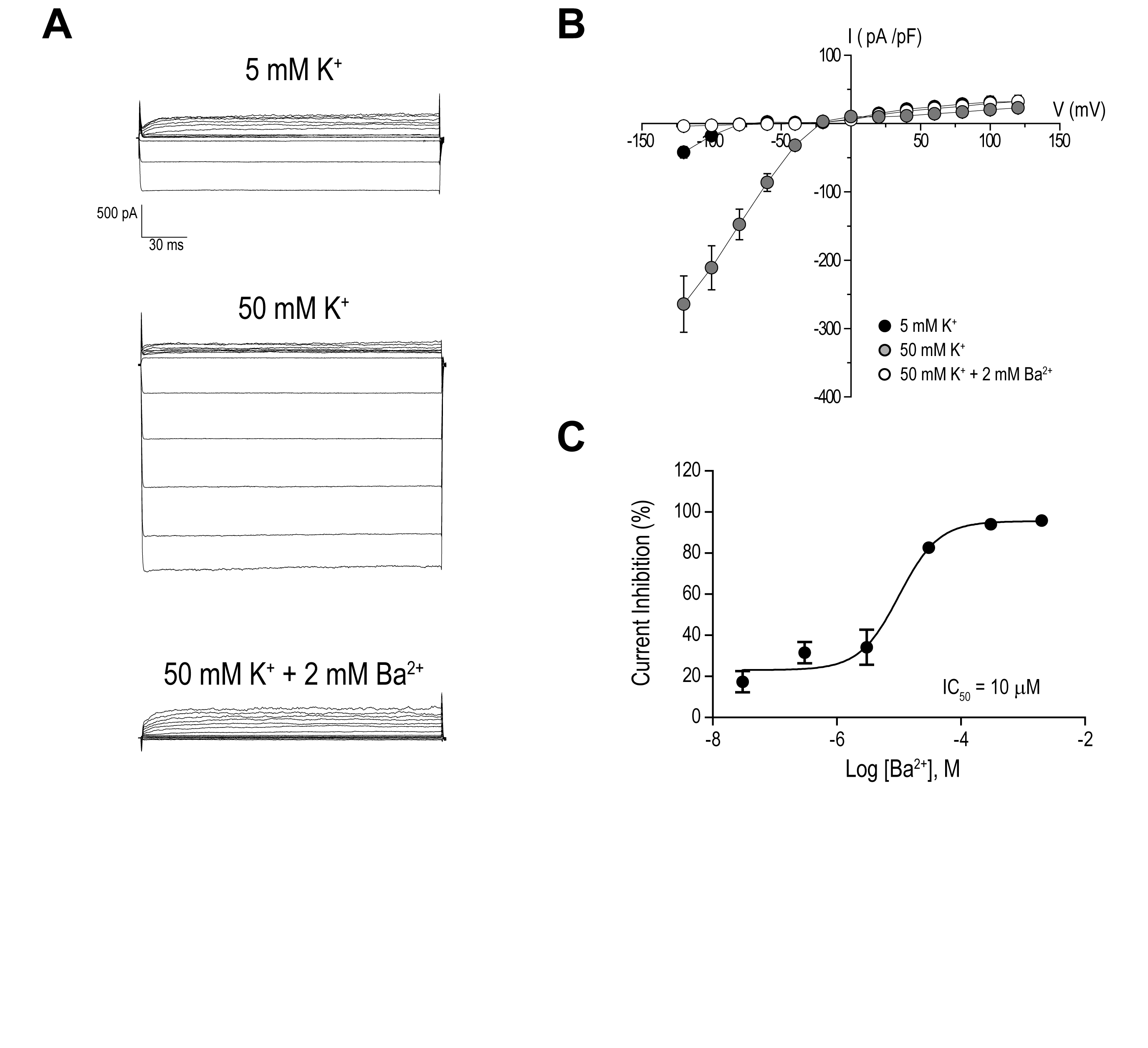

Supplement: Figure S1 — Functional expression of AeKir1 in T-REx-HEK293 cells and its inhibition by barium (Ba2+). (A) Representative current traces recorded from stably transfected cells cultured overnight in the presence of tetracycline to induce channel expression of AeKir1. Recordings were made from a cell superfused with 5 mM K+ (top panel), 50 mM K+ (middle panel), or the control blocker 2 mM Ba2+ in 50 mM K+ (bottom panel). (B) Current (I) -voltage (V) relationships for AeKir1 bathed in 5 mM K+ (dark circle), 50 mM K+ (grey circle), or 50 mM K+ plus 2 mM Ba2+ (white circle). n = 3–7. (C) Concentration-response curve of Ba2+-dependent inhibition of AeKir1 with a 50% inhibition concentration (IC50) of 10 µM. Data are means ± SEM (n = 4–6). (TIF) [file pone.0064905.s001.tif]

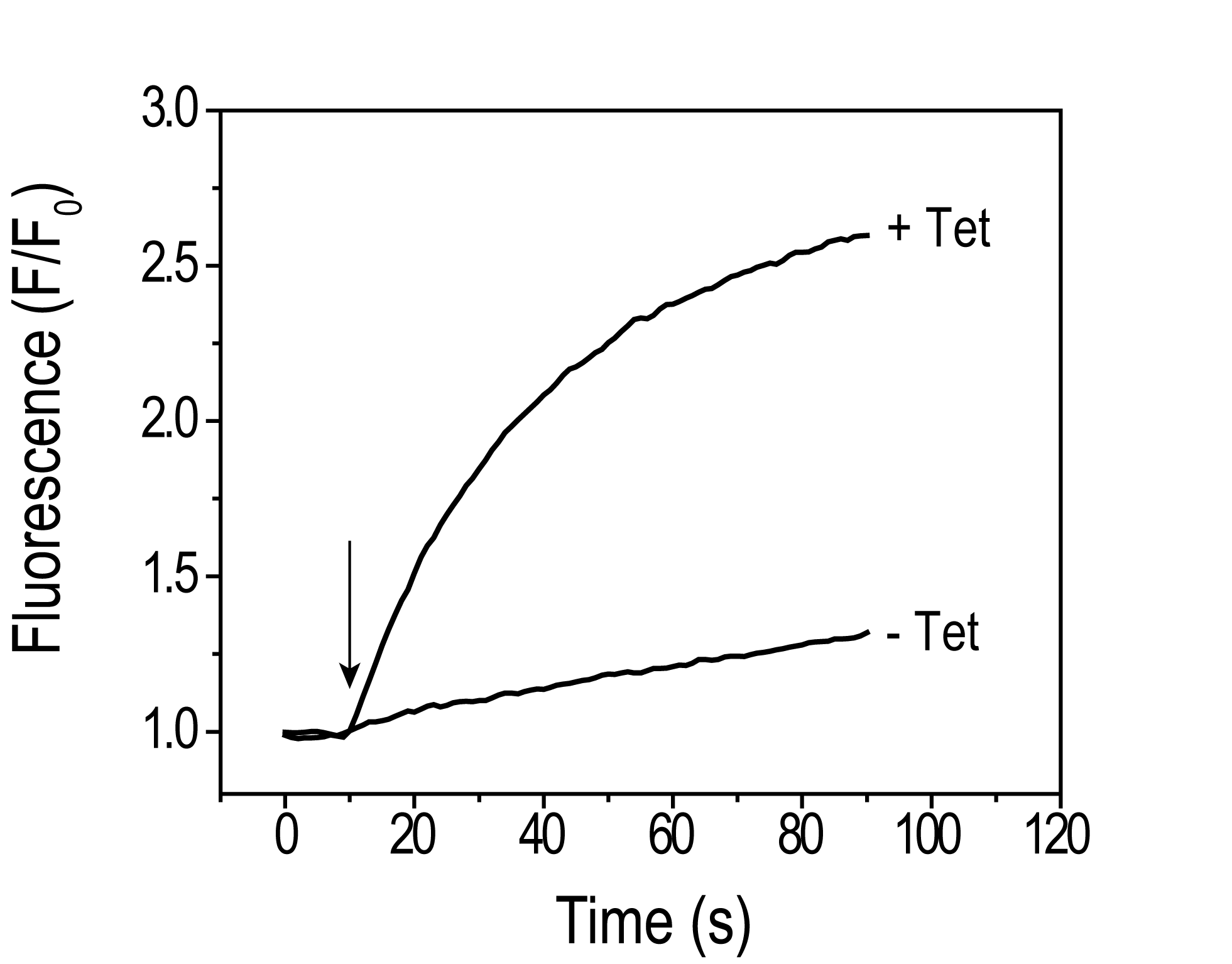

Supplement: Figure S2 — Representative thallium(Tl+)-flux assay in T-REx-HEK293 cells loaded with the FluoZin-2 dye, which fluoresces (F/F0) in the presence of intracellular Tl+. Cells were cultured overnight with tetracycline (+Tet) to induce expression of AeKir1. Cells cultured without tetracycline (−Tet) served as controls . The arrow indicates when Tl+ was added to the extracellular bath. (TIF) [file pone.0064905.s002.tif]

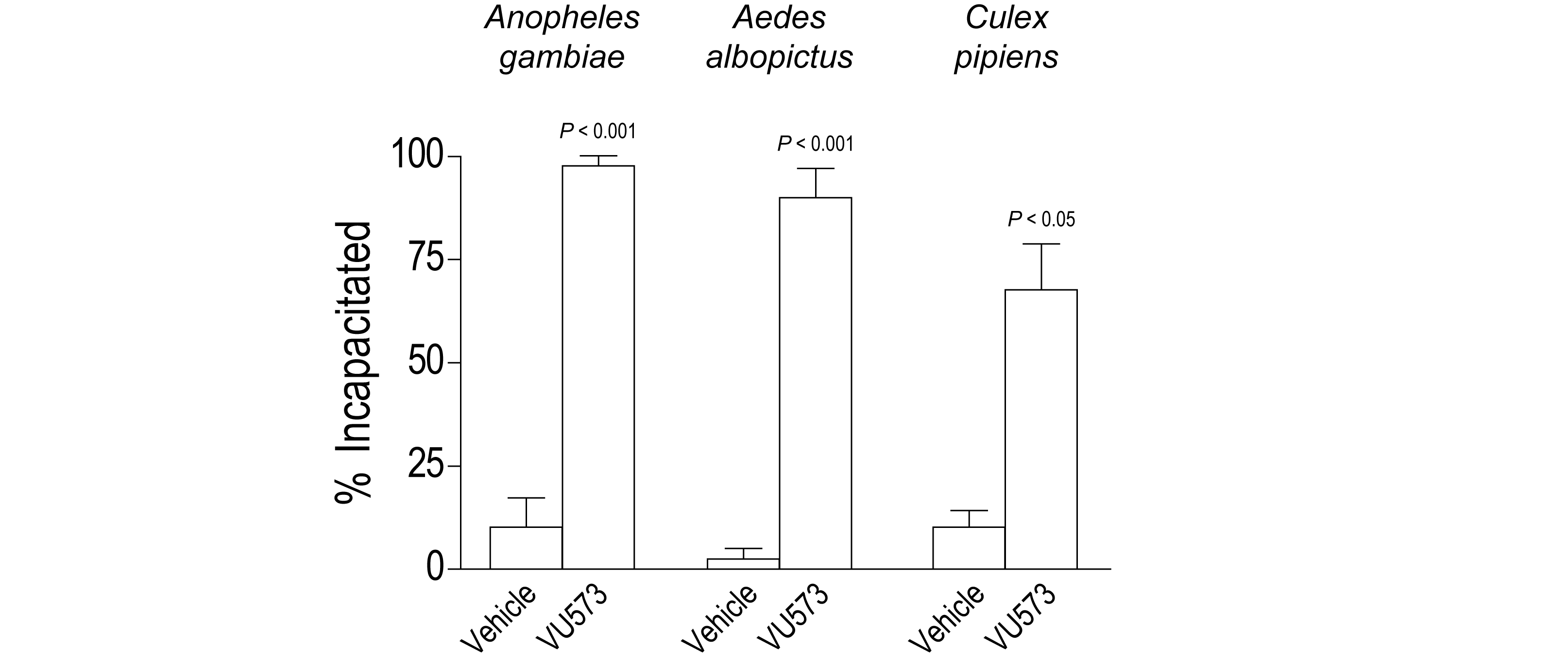

Supplement: Figure S3 — Incapacitating effects of VU573 in three species of mosquitoes. Adult female mosquitoes were injected with Na+- PBS (69 nl) containing the vehicle (15% DMSO) or VU573 (10 mM). ‘% Incapacitated’ refers to the proportion of mosquitoes that are flightless or dead within 24 h after injection. Values are means ± SEM (n = 4 independent trials of 10 mosquitoes). Statistical differences between vehicle and VU573-treated mosquitoes were determined by a paired t-test for each species. Anopheles gambiae is the primary vector of malaria; Aedes albopictus is a vector of emerging arboviruses, such as dengue and Chikungunya fevers; Culex pipiens is a vector of West Nile virus and lymphatic filariasis. (TIF) [file pone.0064905.s003.tif]
