# Supplementary material for: Eliciting Renal Failure in Mosquitoes with a Small-Molecule Inhibitor of Inward-Rectifying Potassium Channels
Source: PLoS One. 2013 May 29;8(5):e64905. doi: 10.1371/journal.pone.0064905 (PMC3666979; doi:10.1371/journal.pone.0064905)
Supplement: Table S1 — Structure-activity relationships for VU573 and its analogs. Values are means ± SEM (n = 1–3 independent Tl+ flux experiments in triplicate). (DOC) [file pone.0064905.s004.doc]

|  | | | | |
| --- | --- | --- | --- | --- |
| **Compound** | **R** | **R’** | **VU#/Barcode** | **IC_50_ (µM)** |
| 1 (VU573) |  | N/A | VU0160573-1/  IC4X | 15 ± 1.2 |
| 2 |  | N/A | VU0403134-1/  IC3Y | 13 ± 3.7 |
| 3 |  | N/A | VU0403131-1/  IC58 | 10 ± 0.0 |
| 4 |  | N/A | VU0340260-1/  IC38 | 20 ± 2.00 |
| 5 |  | N/A | VU0026784-1/  IC3L | 11 ± 2.1 |
| 6 |  | N/A | VU0288495-1  IC39 | 12 ± 0.5 |
| 7 |  | N/A | VU0451348-1/  R6P | 9.4 ± 0.3 |
| 8 |  | N/A | VU0451344-1/  R80 | 14 ± 0.5 |
| 9 (VU342) |  | N/A | VU0451342-1/  R70 | >100 |
| 10 |  | N/A | VU0451341-1/  R7M | 11 ± 1.2 |
| 11 |  | N/A | VU0451340-1/  R5C | 13 ± 3.7 |
| 12 |  | N/A | VU0451339-1/  R56 | 10 ± 0.0 |
| 13 |  | N/A | VU0066224-6/  R7N | 20 ± 2.00 |
| 14 |  | N/A | VU0451336-1/  R5B | 11 ± 2.1 |
| 15 |  | N/A | VU0451333-1/  R5X | 12 ± 0.5 |
| 16 |  | N/A | VU0451337-1/  R5K | 9.4 ± 0.3 |
| 17 |  | N/A | VU0451332-1/  R7B | 14 ± 0.5 |
| 18 |  | N/A | VU0451338-1/  R71 | >100 |
| 19 |  | N/A | VU0451330-1/  R6J | 12 ± 2.1 |
| 20 |  | N/A | VU0451331-1/  RKN | 18 ± 4.0 |
| 21 |  | N/A | VU0451846-2/  R87 | 19 ± 3.0 |
|  | | | | |
| **Compound** | **R** | **R’** | **VU#/Barcode** | **IC_50_ (µM)** |
| 22 |  |  | VU0401333-1/  IC4L | 16 ± 1.5 |
| 23 |  |  | VU0403132-1/  IC48 | >100 |
| 24 |  |  | VU0451343-1/  RJ4 | 18 ± 2.0 |
| 25 |  |  | VU0451335-1/  R5P | 24 ± 0.5 |
| 26 |  |  | VU0451334-1/  R7L | 8.0 ± 0.5 |
|  | | | | |
| **Compound** | **R** | **R’** | **VU#/Barcode** | **IC_50_ (µM)** |
| 27 |  | N/A | VU0467122-1/  1CQH | 7.9 ± 1.1 |
| 28 |  | N/A | VU0467123-1/  15L2 | 33 ± 2.9 |
| 29 |  | N/A | VU0467128-1/  15ND | 8.4 ± 1.1 |
| 30 |  | N/A | VU0467127-1/  15NC | 30 ± 1.1 |
| 31 |  | N/A | VU0467126-1/  15LR | 7.6 ± 1.2 |
| 32 |  | N/A | VU0467125-1/  15KQ | 12 ± 1.1 |
| 33 |  | N/A | VU0467124-1/  15LD | 17 ± 1.2 |
| 34 |  | N/A | VU0026649-2/  1LTK | 26 |
| 35 |  | N/A | VU0027181-2/  1LR8 | 17 |
|  | | | | |
| **Compound** | **R** | **R’** | **VU#/Barcode** | **IC_50_ (µM)** |
| 36 |  |  | VU0469204-1/  1LTW | >100 |
| 37 |  |  | VU0469203-1/  1LTH | >100 |
| 38 |  |  | VU0035129-2/  1LRM | 30 |
| 39 |  |  | VU0469202-1/  1MCF | >100 |
| 40 |  |  | VU0469201-1/  1MDX | 22 |
| 41 |  |  | VU0094518-2/  1MCC | >100 |
| 42 |  |  | VU0469200-1/  1LTU | >100 |
| 43 |  |  | VU0469194-1/  1LTG | >100 |
| 44 |  |  | VU0469195-1/  1LT7 | >100 |
| 45 |  |  | VU0469199-1/  1LU7 | >100 |
| 46 |  |  | VU0469198-1/  1LU6 | >100 |
| 47 |  |  | VU0469197-1/  1LRW | >100 |
| 48 |  |  | VU0469196-1/  1LTT | >100 |
